# The Application of Radiofrequency Waves in Supportive Treatment of Temporomandibular Disorders

**DOI:** 10.1155/2020/6195601

**Published:** 2020-05-06

**Authors:** M. Pihut, M. Górnicki, M. Orczykowska, E. Zarzecka, W. Ryniewicz, A. Gala

**Affiliations:** ^1^Prosthodontics Department, Consulting Room of Temporomandibular Disorders, Jagiellonian University Medical Collage, 4 Montelupich Str., 31-155 Krakow, Poland; ^2^Doctoral Studies, Jagiellonian University Medical College, 12 Św Anny Str., 31-008 Krakow, Poland

## Abstract

In recent years, the number of patients applying for prosthetic treatment due to temporomandibular joint disorders (TMD) has been increasing. The main methods for treating disorders are the use of occlusal splints and physiotherapeutic rehabilitation as supportive treatment. Radio waves are electromagnetic waves with radiation frequency between 3 Hz and 3 THz, used for physiotherapeutic treatment of skeletal muscle relaxation in the range of 3 to 6 MHz. The rehabilitation effect of these waves is based on diathermy by means of high-voltage quick alternating current. *Aim.* The aim of the study was to evaluate the influence of radiofrequency waves on the pain of the masticatory muscles in the course of TMD and the usefulness of these procedures in the supporting treatment of these disorders. *Materials and Methods.* Patients aged 19 to 45 years, of both sexes, reported to the Consulting Room of TMD at the Institute of Dentistry in Krakow to undertake prosthetic treatment of TMD (I a—according to RDC/TMD). Study group (SG) consists of 20 patients who had 10 supportive treatments with radiofrequency currents. In the case of application of radiation to the muscle area, the energy was 20 J to the area of the masticatory muscles, the frequency was 3 MHz, bipolar technique, the duration of the procedure was 10 minutes, and the coupling substance was a gel for ultrasound examinations. The control group (CG) consisted of 20 patients who had 10 supportive treatments with sonophoresis procedures. For the area of masticatory muscles, 0.9 W/cm^2^ treatments were applied, the duty factor was 80%, the treatment time was 10 minutes, and the medical substance was 25% Voltaren gel. *Results.* Analysis of the results of the first clinical examinations (axis I) conducted in both groups shows a homogeneous clinical material and similar results. The second clinical examination revealed improved clinical parameters, but it showed a greater improvement in the SG. In the SG, the mean level of VAS was 6.25, and the extreme values were 5.9–0.14, the median was 2.15, and the standard deviation was 1.54. In the CG, the average value of VAS was 6.20 (peak of 5.2–0.7), the median was 2.4, and the standard deviation was 1.87. *Summary*. The search for new methods of supportive treatment of TMD is an important research direction due to the complex etiology of this disease and the lack of an explicit treatment algorithm. *Conclusion.* The results of our own research clearly indicate that the use of the radiofrequency waves brings pain relief and improvement of clinical parameters to a greater extent than in sonophoresis. It can be a very important new method in supportive treatment of TMD. Research needs to be continued.

## 1. Introduction

In recent years, the number of patients applying for prosthetic treatment due to painful forms of temporomandibular joint disorders has been increasing. Temporomandibular disorders (TMD) include disorders of the masticatory muscles of the stomatognathic system, temporomandibular joints, and the surrounding structures. Functional disorders do not include all diseases associated with musculoskeletal organs, such as inflammatory (multiple sclerosis, tetany, and dermatomyositis), degenerative arthritis, and cancer lesions of the jaws. TMD are often the result of prolonged and excessive work of the mastication muscles and nonphysiological overloads occurring in the temporomandibular jaws and the stomatognathic system associated with excessive muscle tension that sometimes even persists for years [1–5].

The etiology of TMD is complex and multifactorial. There are numerous factors that can contribute to TMD development. They are grinding, clenching as parafunctional activity and abnormal head posture, in conjunction with depression and anxiety, trauma to the facial structures, and effect of acute changes in the occlusal conditions. Iatrogenic factors in all dental specialties also play an important role. Genetic factors and gene polymorphisms and activity of several biogenic amines (serotonin, catecholamine, and glutathione) are also more and more important, and dysregulation of the autonomic nervous system and central pain pathways in TMD is a growing evidence. Some forms of TMD among central sensitization syndromes are a group of pathologies characterized by central morphofunctional alterations [4–9].

The pain form of the disease is manifested by spontaneous pain in the preaural region, accompanied by pain or tenderness of the masticatory muscle. Pain that appears during palpation examination of temporomandibular joints is frequently not related to inflammation of the soft tissue around the temporomandibular joints, as it is described by some authors of the paper [10]. The main cause of this problem is a long-term overload of soft tissue. These factors cause numerous damage to the hard and soft tissues in the masticatory organ, such as excessive overload on the temporomandibular joint structures with subsequent destruction of the joint surfaces, pathological teeth abrasion, tension headaches, or damage to prosthetic restorations. One of the main reasons is concern with psychological status of patients [1, 3, 5–9, 11].

Treatment of TMD is primarily a reversible therapy with the use of occlusion devices called splints and supportive treatment in the form of physiotherapeutic rehabilitation, such as laser biostimulation, sonophoresis, and iontophoresis with the use of analgesics or hyaluronic acid, manual therapy, kinesiotherapy, magnetoledotherapy, and poisometric muscle relaxation. The main aim of this treatment is to reduce the increased tension of masticatory muscles and to eliminate pain in the muscles and joints [1, 2, 10–15].

Radio waves are electromagnetic waves with radiation frequency between 3 Hz and 3 THz, used for physiotherapeutic treatment of striated muscle relaxation in the range of 3 to 6 MHz. The use of radiofrequency waves is becoming more and more widely used in the relaxation therapy of skeletal muscles in orthopaedics or traumatology and is becoming an important method used in rehabilitation. The mechanism is based on oscillation of electric current in the range of two to three thousand per second, transforming it into thermal energy released around the tip of the electrode that heats the medium (epidermis, dermis subcutaneous tissue, muscles, or joints). The rehabilitation effect of these waves is based on diathermy by means of high-voltage quick alternating current. The conversion of energy causes the generation of heat, which warms muscles to about 45°C. Treatments are used mainly to improve blood supply to the irradiated tissue, stimulate regenerative processes, reduce muscle tension, and obtain an analgesic effect. The heat generated inside the tissue results within the tissue in dilatation of blood vessels, improvement of tissue blood circulation, stimulation of metabolic processes, acceleration of tissue absorption processes, increase in the number of leukocytes, reduction of muscle tension, reduction of excitability of the musculoskeletal-neural system, and analgesic effect [16–21].

Currently, due to their proven effectiveness and lack of side effects, radiofrequency currents are used to treat back pain, trigeminal nerve neuralgia, and spinal pain, especially in the lumbar region and joints. Literature data also indicate the possibility of using radio waves as an alternative procedure for the treatment of chronic cluster headaches. Contraindications for the use of these procedures include pregnancy, presence of metal implants in the body, cancer, epilepsy, skin diseases, open wounds, acute thyroid diseases, hyperthyroidism, and Sudeten disease. In addition, attention should be paid to spot atrophy, vein inflammation, cataract, sensory disorders, psychosis, electronic implants, pacemaker, and tuberculosis [19–26].

Among the growing number of publications on the beneficial effects of RF on muscle tissues, there are only a single data on its beneficial therapeutic effects in patients with joint disorders and head and neck ailments, which were the inspiration for this study [16, 17].

The aim of the study was to evaluate the influence of radiofrequency waves on the masticatory muscles in the course of TMD of the stomatognathic system, and thus the usefulness of these procedures in the physiotherapeutic rehabilitation as a supportive treatment.

## 2. Materials and Methods

The research obtained approval from Ethics Committee of Jagiellonian University (no. 1072.6120.116.2018) (Clinical trials no. 1072.6120.116.2018).

The study group SG (20 patients) and control group II (20 patients) consisted of patients, aged 19 to 45 years, of both sexes, who came to the Consulting Room of Temporomandibular Joint Disorders in the Institute of Dentistry in Krakow to undertake prosthetic treatment of pain form of temporomandibular disorders with the dominant muscle component. All patients qualified for this study were generally healthy and were treated between April 2018 and December 2018. The SG consists of 20 patients, who had 10 procedures of radiofrequency currents, as a supportive treatment in the period of preparing the splint. In the case of application of radiation to the muscle area, the energy was 20 J, the frequency was 3 MHz, bipolar technique, the duration of the procedure was 10 minutes, and the coupling substance was a gel for ultrasound examinations.

The CG consisted of 20 patients who had 10 sonophoresis procedures as supportive treatment. For the area of mastication muscles, 0.9 W/cm^2^, treatments were applied, the duty factor was 80%, the treatment time was 10 minutes, and the medical substance was 25% Voltaren gel.

All physiotherapeutic treatments were performed every day except Sundays.

All patients were subjected to a medical interview and physical examination of function of the masticatory organ according to diagnostic criteria for temporomandibular disorders (DC/TMD axis I and axis II) for clinical application [1–3, 12].

Factors of inclusion in the study were patient's consent to participate in the study, TMD (I a-according to RDC/TMD), the required age range, a good general state of health, which means that patients did not suffer from medical conditions and no contraindications to the use of radiofrequency currents and sonophoresis (pregnancy, the presence of metal implants, epilepsy, phlebitis, skin diseases, open wounds, heard pacemaker, and sensory disturbances), and full dental arches.

Factors for exclusion from the research were as follows: patient's will to resign from participation in research and the appearance of general diseases or occurrence of a contraindication to the use of evaluated therapy (radiofrequency and sonophoresis) that prevent participation in the project, occurrence of trauma, or local inflammations.

During the study, we analyzed information concerned with general health and presented chief complaints to elimination of general diseases, which is the exclusion criterion. Through a medical interview, detailed information about the current general health condition was revealed (including information about chronic and hereditary diseases); the examining physician obtained information about patient's surgical treatment and head trauma history within the last 6–8 months, and the current medications were taken on daily basis. In this context, particular attention was given to muscle and joint conditions, with a focus on multiple sclerosis, osteoporosis, fibromyalgia, trigeminal neuralgia, hormonal disorders, and autoimmune diseases for differential diagnosis.

Diagnostic criteria RDC/TMD axis I procedures for examination of the stomatognathic system were conducted, with particular regard to range of maximal opening and the path of mandibular motion and its deviation, scope of lateral and forward motion, the nature and intensity of the perceived pain, trigger points and discomfort of temporomandibular joints and masticatory muscles, which occurred prior to treatment and radiation of pain, the type of sound symptoms (clicking and popping), volume, and the phase of the movement of lowering and lifting the jaw, during which there was found tension-type headache (Table 1).

A clinical examination of the temporomandibular joint consists of palpation and auscultation test; palpation was performed simultaneously on both sides of the face by exerting the force of around 450 g per area of a square centimeter. The individual palpation force was measured using electronic kitchen scales. Auscultation was performed with the aid of a double-tube stethoscope.

In the examination in axis II, the most important was evaluation made by patients of the present intensity of pain of mastication muscles with the use of VAS scales (axis II, p. 7).

Clinical examination according to RDC/TMD diagnostic criteria was carried out in both groups twice, before and after 10 physiotherapeutic supportive procedures.

The occlusal splints were applied after physiotherapeutic rehabilitation.

## 3. Statistical Analysis

For statistical analysis, software STATISTICA (Tibco) v. 13.3 was used. A *p* value less than 0.05 was deemed as statistically significant. Significant statistical differences in SG and CG were determined by the Mann–Whitney test. Nonparametric methods were used as normality assumptions which were violated with regard to variables' distribution (the Shapiro–Wilk test was calculated beforehand for checking distributional adequacy).

Additionally, in our research, descriptive, basic statistical analysis was calculated including variables' mean values, standard deviations, minimal and maximal values, skewness, and kurtosis.

## 4. Research Results and Discussion

The research involved 40 patients, ranging in age from 19 to 45 years, divided into two groups of 20 people. The SG included 14 women and 6 men (mean age 26), and the CG included 12 women and 8 men (mean age 28). Analysis of the results of the first clinical examinations (axis I) conducted in both groups shows a homogeneous clinical material (range of motion of the jaw, abnormal range and symmetry of movement of the jaw, pain of the muscles, parafunctions, and difficulty in chewing foods). The second clinical examination conducted in both groups revealed improved clinical parameters, but it showed a greater improvement in the SG (Table 1). Table 1 presents the percentage amount of symptom severity among patients in both groups as well for easier distinction of results.

**Figure 1:**
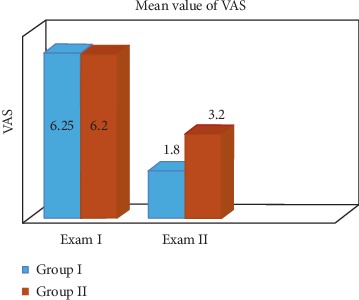
Mean values of the VAS score in two groups obtained in examination I and II (axis II).

Assessment of the intensity of currently felt pain (axis II, point 7) in the SG at the first examination revealed the mean level of masticatory pain was 6.25, and the border values were 4.0–9.0, the median was 6, and the standard deviation was 1.371. In the CG, the average value of intensity of pain was 6.20 (peak of 5.2–0.7), the border values were 3.0–9.0, the median was 6, and the standard deviation was 1.735. In the second examination (after supportive treatment executing), the mean value of pain punctuation in the SG was 1.85, border values were 1.0–3.0, median was 2.0, and standard deviation was 0.489. In the CG, the results are as follows: mean value of the punctuation was 3.20, the border values were 1.0–6.0, the median was 3.0, and the standard deviation was −1.735. The results of average pain scores differed between groups in a statistically significant way in favor of SG because the *p* value was 0.0146. The results and statistical calculations are presented in Table 2 and graphically presented in Figure 1.

Statistical analysis of intensity of pain shows that the results between the first examination of both groups did not differ in a statistically significant way (homogeneity of groups), but comparison between the first and second examination in both groups differs in a statistically significant way because *p* value is less than 0.05 (see Table 3).

The results of the preliminary study indicate a positive effect of the method being evaluated on the improvement of clinical parameters (tension of the muscles, difficulty in chewing, and mandibular deviation during opening motion) and a decrease in the intensity of pain in the mastication muscles after the supporting treatment of temporomandibular joint disorders with radiofrequency waves and sonophoresis. The better result was obtained in the SG, which is included in Table 1.

One of the main etiological factors of TMD is occlusive parafunctions, like harmful movement habits performed by the patient which do not serve the purpose of physiological functions (eating, singing, speaking, and sifting) [14]. They cause long-term systolic work of the muscles lifting the mandible, thus generating not only a pathological increase in the mastication muscles but also overloads in the temporomandibular joints. Therefore, one of the main objectives of TMD-supportive treatment is to relax the masticatory muscles and eliminate trigger points in the muscles responsible for pain and tenderness of the muscles [1–4, 10, 11].

Treatment of TMD due to the complex etiology of this disease is a difficult task and requires in-depth analysis of the reported symptoms and results of the clinical examination. In supportive (symptomatic) treatment, each new method of proven efficacy is a valuable complement to basic therapeutic methods using occlusal splints. The literature data indicate the beneficial effect of radiofrequency radiation on transversally striated (skeletal) muscles and provide grounds to believe that the procedures evaluated may constitute a new method of TMD support treatment. Raising the temperature in the muscles combined with a gentle massage of the device head causes relaxation of tense muscles, overloaded with prolonged systolic work (as a result of occlusal parafunctions). The results of own preliminary research confirm this thesis [16, 21–23].

Many authors emphasize the beneficial effect of RF on damage to many structures in the body, such as trigeminal neuralgia, treatment of knee injuries, and shoulder pain of various origins [17–25]. Van Tilburg et al. in double-blind studies showed a beneficial effect of RF on spinal pain in the lumbar region [24]. Li et al. [17] evaluated the influence of several therapeutic methods (biostimulation laser, shock wave, ultrasounds, and radio frequency) on the effectiveness of pain treatment in case of heel tendonitis. The evaluation of RF effectiveness was positive. Fang et al. [18] and Gou et al. [22] emphasized that radiofrequency radiation is imperative and effective in the treatment of vulvar nerve neuralgia. Kwak et al. evaluated the effectiveness of cervical radicular pain treatment with RF. Their results show that RF is effective for alleviating cervical radicular pain, which was unresponsive to oral medications, physical therapy, or epidural steroid injection [19]. Comparison of the analgesic effects of pulse radiofrequency and cryoablation in rabbits with mental nerve neuropathic pain made by Canpolat et al. was positive for RF [20]. Choi et al. emphasized the opinion that radiofrequency treatment relieves chronic knee osteoarthritis pain which they proved in a double-blind randomized controlled trial [26].

Those positive results of investigations of many authors give hope that the use of radiofrequency waves will be a new method for effective supporting treatment of temporomandibular joint disorders, which is very important due to complex etiology of these disorders and therapeutic difficulties.

Summary: the search for new methods of supportive treatment of TMD is an important research direction due to the complex etiology of this disease and the lack of an explicit treatment algorithm.

## 5. Conclusion

The results of own research clearly indicate that the use of the radiofrequency waves brings pain relief and improvement of clinical parameters. It can be a very important new method in supportive treatment of temporomandibular joint disorders. Research needs to be continued.

## Figures and Tables

**Table 1:** Some symptoms of TMD obtained during I and II clinical examination of the stomatognathic system (axis I and II) conducted in both groups.

Symptoms of TMD			Group I		Group II		Group I		Group II	
			Exam 1		Exam 1		Exam 2		Exam 2	
			*N*	%	*N*	%	*N*	%	*N*	%
Pain of temporomandibular joint	Single		0	0	0	0	0	0	0	0
	Bilaterally		0	0	0	0	0	0	0	0
Pain of masticatory muscles presence during lowering the jaw			16	80	16	80	4	20	6	30
Pain of the masticatory muscles triggered by palpation			16	80	16	80	8	40	11	55
Pain of the masticatory muscles associated with the border position of the jaw			15	75	34	17	6	30	8	40
Spontaneous masticatory muscle pain			14	70	17	85	6	30	11	55
Radiation of pain			9	45	10	50	3	15	5	25
Sounds in temporomandibular joints	Single	R	0	0	0	0	0	0	0	0
		L	0	0	0	0	0	0	0	0
	Bilaterally		0	0	0	0	0	0	0	0
Subjective feeling of increased tension in muscles			15	75	16	80	4	20	8	40
Limited opening of the lower jaw			0	0	0	0	0	0	0	0
Limited lateral mandibular movements			0	0	0	0	0	0	0	0
Mandibular deviation during opening			11	55	7	35	6	30	3	15
Difficulty in chewing			16	80	15	75	5	25	8	40

**Table 2:** The result of statistical analysis of masticatory muscles pain evaluation.

Examinations	*X* ± SD	Min.-Max.	Median	Mann–Whitney *U* test
Group I, exam 1	6.25 ± 1.371	4.0–9.0	6.0	—
Group II, exam 1	6.20 ± 1.735	3.0–9.0	6.0	—
Group I, exam 2	1.85 ± 0.489	1.0–3.0	2.0	—
Group II, exam 2	3.20 ± 1.735	1.0–6.0	3.0	—
Exams 1 and 2 within group I	4.05 ± 2.449	1.0–9.0	3.5	*p* ≤ 0.001
Exams 1 and 2 within group II	4.70 ± 2.289	1.0–9.0	5.0	*p* ≤ 0.001
Group I and group II: exam 1	6.23 ± 1.544	3.0–9.0	6.0	*p*=0.9559
Group I and group II: exam 2	2.53 ± 1.432	1.0–6.0	2.0	*p* ≤ 0.001

**Table 3:** The radiofrequency parameters used in the treatment of the patients in groups SG and CG.

Group	Age	*N*	Medical intervention	Amount of procedures/time of duration	Parameters of the procedure
SG	19–45	20	Radiofrequency	10/10	20 J
					3 MHz
					Bipolar technique
CG	19–45	20	Sonophoresis	10/10	0. 9 W/cm^2^
					80% duty factor
					25% Voltaren gel

## Data Availability

The data used to support the findings of this study are available from the corresponding author upon request.
